# Exploring the role of miR-200 family in regulating CX3CR1 and CXCR1 in lung adenocarcinoma tumor microenvironment: implications for therapeutic intervention

**DOI:** 10.1038/s41598-023-43484-1

**Published:** 2023-09-28

**Authors:** Archana Sharma, Prithvi Singh, Rishabh Jha, Saleh A. Almatroodi, Faris Alrumaihi, Arshad Husain Rahmani, Hajed Obaid Alharbi, Ravins Dohare, Mansoor Ali Syed

**Affiliations:** 1 Translational Research Lab, Department of Biotechnology, Faculty of Natural Sciences, Jamia Millia Islamia, New Delhi, 110025 India; 2 Centre for Interdisciplinary Research in Basic Sciences, Jamia Millia Islamia, New Delhi, 110025 India; 3 Department of Medical Laboratories, College of Applied Medical Sciences, Qassim University, 51452 Buraydah, Saudi Arabia

**Keywords:** Cancer, Computational biology and bioinformatics, Genetics, Systems biology, Biomarkers

## Abstract

Lung adenocarcinoma (LUAD) is the most common malignant subtype of lung cancer (LC). miR-200 family is one of the prime miR regulators of epithelial-mesenchymal transition (EMT) and worst overall survival (OS) in LC patients. The study aimed to identify and validate the key differentially expressed immune-related genes (DEIRGs) regulated by miR-200 family which may serve for therapeutic aspects in LUAD tumor microenvironment (TME) by affecting cancer progression, invasion, and metastasis. The study identified differentially expressed miRNAs (DEMs) in LUAD, consisting of hsa-miR-200a-3p and hsa-miR-141-5p, respectively. Two highest-degree subnetwork motifs identified from 3-node miRNA FFL were: (i) miR-200a-3p-*CX3CR1*-*SPIB* and (ii) miR-141-5p-*CXCR1*-*TBX21*. TIMER analysis showed that the expression levels of *CX3CR1* and *CXCR1* were significantly positively correlated with infiltrating levels of M0-M2 macrophages and natural killer T (NKT) cells. The OS of LUAD patients was significantly affected by lower expression levels of hsa-miR-200a-3p, *CX3CR1* and *SPIB*. These DEIRGs were validated using the human protein atlas (HPA) web server. Further, we validated the regulatory role of hsa-miR-200a-3p in an *in-vitro* indirect co-culture model using conditioned media from M0, M1 and M2 polarized macrophages (THP-1) and LUAD cell lines (A549 and H1299 cells). The results pointed out the essential role of hsa-miR-200a-3p regulated *CX3CL1* and *CX3CR1* expression in progression of LC TME. Thus, the study augments a comprehensive understanding and new strategies for LUAD treatment where miR-200 family regulated immune-related genes, especially chemokine receptors, which regulate the metastasis and invasion of LUAD, leading to the worst associated OS.

## Introduction

LUAD is a common malignant subtype of NSCLC, accounting for more than $40{\%}$ of LC cases worldwide^1^. It is a multifactor and multistage process associated with multiple genes where the absolute risk of distant metastasis is very high at every stage of disease^2^. This primarily enhances the disease’s systemic threat, leading to poor prognosis, higher recurrence rates, and lower OS of patients^3^.

miRNAs are a class of small ($19-25$ nucleotides), highly conserved, ncRNA molecules that can modulate various molecular mechanisms by inhibiting translational or mRNA degradation. miR-200 family (i.e., miR-200a, miR-200b, miR-200c, miR-141, and miR-429) are among the crucial miRNAs which primarily regulate EMT in LC. It can be divided into two groups based on single nucleotide seed sequence difference—(i) miR-200b, miR-200c, and miR-429 (AAUACUG) and (ii) miR-200a and miR-141 (AACACUG). Even after being recognized as a crucial tumor-associated miRNA, the precise molecular mechanism involved in miR-200 family-mediated LC progression and metastasis is unknown mainly due to the controversial results as tumor suppressor or oncogenic levels in serum and/or tissue as well as at various stages of cancer. The miR-200 family has been associated with poor prognosis and worst OS in NSCLC patients, especially LUAD^4,5^. Therefore, further insight into the miR-200 regulated mechanisms for developing and progressing LUAD is required.

TME is a key player in tumor progression and metastasis, which may lead to identifying novel targets and developing novel therapies. Immune cells, a primary part of TME, play a critical role in tumor growth and development^6^. Cellular cross-talk between tumor cells and their TME, which consists of CAFs, leukocytes, various infiltrating immune cells, and non-cell components of the ECM, contributes to cancer progression. This is facilitated by various soluble factors like growth factors and chemokines^7^. Chemokine receptors are extensively expressed on tumor and TME cells, facilitating various processes such as tumor cell survival, angiogenesis, vascular permeability, leukocyte recruitment, immune suppression, tumor cell adhesion, proliferation, EMT, and metastasis^8^.

The present study identified the DEGs and DEMs between tumor and normal tissues based on TCGA-LUAD cohort followed by DEIRGs identification. Since our study focused on the miR-200 family, hence a closed 3-node FFL was created, which showed regulation of these DEIRGs (chemokine receptors) by miR-200 family and corresponding TFs. The OS of LUAD patients affected by miR-200 family-associated FFL hub elements was depicted using KM plots. Furthermore, the TIMER database revealed the correlation between the expression of miR-200 family-associated DEIRGs and tumor-infiltrating immune cells in LUAD patients. The expression results of miR-200 targeted DEIRGs obtained in our study were validated with HPA database. To further validate the regulatory role of miR-200a-3p in TME, the expression of *CX3CR1* and *CX3CL1* were studied in miR-200a-3p transfected LUAD cell lines (A549 and H1299 cells) which were cultured in conditioned media from M0, M1 and M2 polarized macrophages (THP-1 cell line).

Therefore, our study emphasizes on the critical role of miR-200 family-targeted DEIRGs which have an essential role in TME during metastasis. Targeting these miR-chemokine receptor axes might have therapeutic potential in treating LUAD.

## Materials and methods

### TCGA RNA-seq data extraction and DEA

mRNA HTseq and miRNA-seq count data of TCGA-LUAD were retrieved from the UCSC Xena browser (https://xenabrowser.net/)^9^. Back-log-transformation and cross-checking of the mRNA-seq and miRNA-seq LUAD cohort samples with TCGA-GDC^10^ data portal was performed as discussed previously^11,12^. Pre-processing (i.e., normalization and ${\mathrm{log}}_{2}$ transformation) of mRNA-seq and miRNA-seq cohorts was performed as discussed previously^11,12^. Batch correction of pre-processed values was performed as discussed previously^12^. Ensembl IDs to their corresponding HGNC symbol(s) mapping in the mRNA-seq cohort was performed as discussed previously^12^. Duplicate genes were handled as discussed previously^13–18^. Limma package^19^ was used for the identification of DEGs and DEMs corresponding to a threshold of $\left|{\mathrm{log}}_{2}\left(\mathrm{fold change}\right)\right|>2$ and $\mathrm{BH}-\mathrm{ p}-\mathrm{value}<0.0001.$

### Enrichment and PPIN analyses of LUAD-associated immune genes

The ImmPort (https://immport.niaid.nih.gov)^20^ is a critical repository for immunology-associated clinical and molecular data. The overlapping genes between human IRGs acquired from ImmPort and our filtered DEGs were categorized as the DEIRGs. Pathway and GO term enrichment analyses of our DEIRGs were performed using the ReactomePA package^21^. A $\mathrm{q}-\mathrm{value }<0.0001$ was used as the preferred cutoff for selecting significantly enriched pathway and GO terms. The sequential steps of PPIN formation and cluster selection were performed as discussed previously^22^. The genes present in the topmost-scoring PPIN cluster were regarded as the hub DEIRGs, respectively.

### miRNA-mRNA-TF regulatory network construction and ROC curve analysis

Significant human TFs interacting with our hub DEIRGs were acquired from ChEA v3.0 database (https://maayanlab.cloud/chea3/)^23^ corresponding to a $p-\mathrm{value}\le 0.001$. miRNAs which were interacting with our hub DEIRGs and TFs were extracted as discussed previously^15^. The miRNAs overlapping with DEMs were retained and regarded as validated miRNAs. Sequential steps of 3-node miRNA FFL formation and visualization were performed as discussed previously^11,15^. ROC curve analysis was performed to assess the diagnostic sensitivity of miR-200 family-associated hub mRNAs in LUAD patients. The AUC assessed the diagnostic values.

### Tumor immune infiltration analysis

TIMER (http://timer.cistrome.org/)^24^ is a web resource for systematically analyzing the immune infiltration across various cancer types. Using TIMER, we assessed the correlation between the expression of miR-200 family-associated hub mRNAs and tumor-infiltrating immune cells in LUAD patients. Spearman correlation was utilized to evaluate the statistical significance. The gene expression levels were ${\mathrm{log}}_{2}$ RSEM expressed.

### Validation of miR-200 family-associated hub DEIRGs using HPA and GEO

The protein expression levels of hub DEIRGs targeted by miR-200 family members were determined using the HPA database (https://www.proteinatlas.org/)^25–29^ in normal and LUAD tissues. NCBI-GEO (https://www.ncbi.nlm.nih.gov/geo/)^30^ was inquired utilizing “LUAD” and “lung adenocarcinoma” as keywords for extracting mRNA expression profiles. The search results were filtered as per the inclusion and exclusion criteria mentioned previously^31^. Sequential steps of batch correction, gene mapping, duplicate genes removal, DEGs identification were performed as discussed previously^31^. The presence of hub DEIRGs targeted by miR-200 family members was cross-checked in the DEGs list.

### Survival analysis of miR-200 family-associated FFL hub items

KM plots showing the OS of 3-node miRNA FFL-associated subnetwork motif items were displayed as per parameters discussed previously^32^ via KM plotter database (https://kmplot.com/analysis/)^33^ corresponding to TCGA-LUAD cohort patient samples.

### Cell culture, macrophage differentiation and transient transfection

Human LUAD cell lines-A549 and H1299 along with THP-1 (Human monocytes) were procured from NCCS, Pune, India. These cell lines were cultured in RPMI1640 (Cat # 61,870,036, Gibco, Waltham, MA, USA), $1{\%}$ antibiotic–antimycotic (Cat # 15,240,096, Gibco, Waltham, MA, USA) at $37\, ^\circ \mathrm{C}$ and $5{\%}$ ${\mathrm{CO}}_{2}$. Differentiation of THP-1 cells were done by using $5\,\mathrm{ ng}/\mathrm{mL}$ PMA (P8139, Sigma, Saint Louis, Missouri 63113, USA). They were stimulated using LPS ($100\,\mathrm{ ng}/\mathrm{ml}$) and IL-4 ($10\,\mathrm{ ng}/\mathrm{mL}$, Cat # I4269, Sigma Aldrich, Bangalore, India) for $24\,\mathrm{ h}$ for obtaining M1 and M2 macrophage cell population, respectively. A differentiated but non-stimulated macrophage population was termed as M0. Adherent cells were washed and cultured with serum-free RPMI medium for $24\,\mathrm{ h}$, then the resulting macrophage-conditioned medium from M0, M1 and M2 macrophage population was collected and clarified by centrifugation at $13,000$ rpm at $4\, ^\circ \mathrm{C }$ for $5\,\mathrm{ min}$. A549 and H1299 cells were transiently transfected with $30\,\mathrm{ pmol}/\mathrm{mL}$ scrambled miRNA mimic (Qiagen, Hilden, Germany) and $30\,\mathrm{ pmol}/\mathrm{mL}$ miR-200a-3p mimic (Qiagen, Hilden, Germany) using lipofectamine 3000 (Invitrogen, Waltham, MA, USA) for $24\,\mathrm{ h}$. These scrambled and miR-200a-3p transfected cells from both A549 and H1299 cell lines were culture with $1:5$ concentration of conditioned media from M0, M1 and M2 macrophages for $18\,\mathrm{ h}$. The treated cells were further subjected to RNA isolation.

### qRT-PCR analysis

Total RNA was isolated from M0, M1 and M2 THP-1 macrophages and scrambled/miR-200a-3p transfected A549 and H1299 cells which were treated with M0/M1/M2 macrophage-conditioned media using TRIzol reagent (Ambion, CA, USA) following the manufacturer’s protocol. The iScript cDNA synthesis kit (Bio-Rad, Hercules, CA, USA) was used to reverse-transcribe $1\,\upmu \mathrm{g}$ of this isolated RNA. qRT-PCR analyses for quantifying mRNA of *CX3CR1*, and its ligand gene *CX3CL1* and actin were performed by using iTaq Universal SYBR Green Supermix (Bio-Rad, Hercules, CA, USA). PCR using actin as an endogenous control was performed using 7900HT Fast Real-time PCR System (Applied Biosystems, Waltham, MA, USA). Relative quantification from real-time data is presented based on the calculation of ${2}^{-\Delta \Delta \mathrm{Ct}}$. The primer sequences used are *CX3CR1*-forward primer 5′-AAATACCCCATCATTCATGC-3′, reverse primer 5′-TTGTTCCAAACGTTTCTAGG-3′, CX3CL1-forward primer 5′-AGATACCTGTAGCTTTGCTC-3′, reverse primer 5′-TCTCGTCTCCAAGATGATTG-3′, Actin- forward primer 5′-AGCACAGAGCCTCGCCTT-3, reverse primer 5′- CATCATCCATGGTGAGCTGG-3′.

### Cell proliferation assay

A549 and H1299 cells were harvested, seeded in $96$-well plates ($100\ \upmu \mathrm{ L}$/well), and cultured for $24\,\mathrm{ h}$. They were both transiently transfected with $30\,\mathrm{ pmol}/\mathrm{mL}$ scrambled miRNA mimic (Qiagen, Hilden, Germany) and $30\,\mathrm{ pmol}/\mathrm{mL}$ miR-200a-3p mimic (Qiagen, Hilden, Germany) by using lipofectamine 3000 (Invitrogen, Waltham, MA, USA) for $24\,\mathrm{ h}$. A549 and H1299 cancer cells were then cultured in a $20{\%}$ conditioned medium from M0/M1/M2 macrophages in RPMI complete medium. MTT assay was performed to evaluate cell proliferation on completion of $24\,\mathrm{ h}$ ($\mathrm{N}=3$ for each group).$5\mathrm{ \mu L}$ of MTT solution (Sigma-Aldrich, St Louis, MO, USA) ($5\,\mathrm{ mg}/\mathrm{mL}$) were added to each well, and incubated for $3-4\mathrm{ h}$ at $37\, ^\circ \mathrm{C}$ and $5{\%}$ ${\mathrm{CO}}_{2}$. The supernatant was discarded, then $100\,\mathrm{ \mu L}$ of DMSO was added to each well to dissolve the formazan crystal violet. The absorbance was measured at $570\,\mathrm{ nm}$. Percent proliferation was calculated for all the groups.

### Statistical analysis

All the experiments were repeated thrice independently, and the values obtained were expressed as the means $\pm$ SEM. Data obtained from the study were analyzed by using GraphPad Prism version 7.0. Significance levels between two corresponding groups under comparison were checked using students t-test. $p-\mathrm{value}<0.05$ was considered to be statistically significant.

## Results

### RNA-seq data extraction and DEA

mRNA and miRNA LUAD cohorts comprised $444$ samples ($433$ tumor and $11$ healthy normal samples). After batch correction, gene mapping, and duplicacy removal, we were left with $19193$ genes. Similarly, after removing low-count miRBase IDs, we were left with $1355$ miRBase IDs out of $1881$. Using limma, we identified a total of $1053$ DEGs and $46$ DEMs corresponding to the abovementioned threshold i.e., $\left|{\mathrm{log}}_{2}\left(\mathrm{fold change}\right)\right|>2$ and $\mathrm{BH}-p-\mathrm{value}<0.0001$. Figure S1A shows an annotation heatmap plot of top $10$ down and top 10 upregulated DEGs. The sample annotation bar at top of the heatmap clearly illustrates more female samples ($54.72{\%}$) than male samples ($45.72{\%}$). The heatmap shows healthy normal samples clustered distinctly from tumor samples. Chromosome number $10$ was populated with the highest number of DEGs (*SFTPA1*, *SFTPA2*, *GDF10*, and *SFTPD*) and all of these were downregulated.

### Enrichment and PPIN modular analyses of DEIRGs

**Figure 1:**
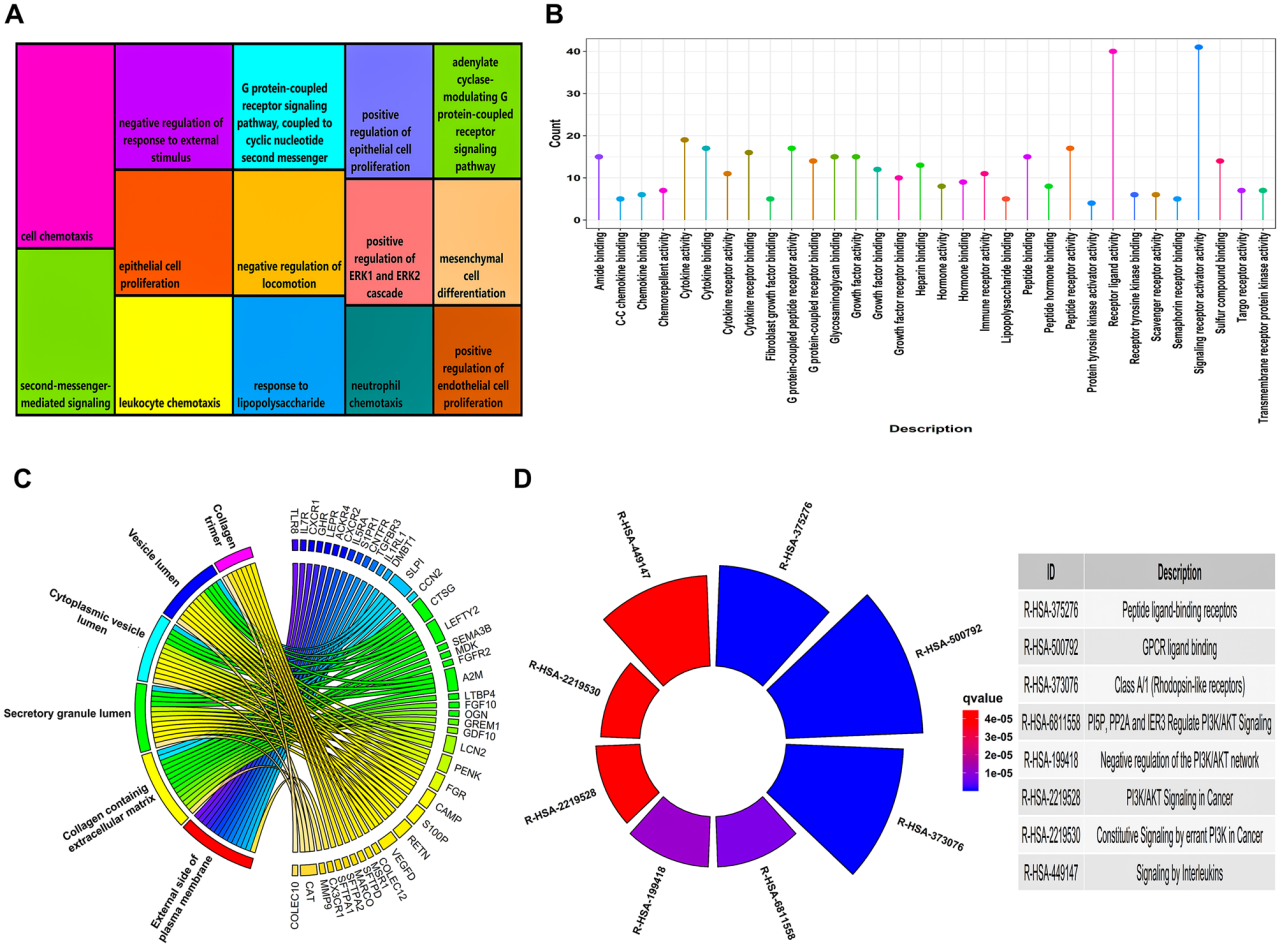
(**A**) Treemap chart illustrating significant GO-BP terms associated with DEIRGs. Each rectangular shape represents individual terms and their sizes vary according to the number of genes present in them. All the rectangles have a unique color signifying the distinct $14$ BP terms. The term “cell chemotaxis” has the highest number of genes present in it, i.e., $27$. While the terms “neutrophil chemotaxis” and “positive regulation of endothelial cell proliferation” has the lowest number of genes present in it, i.e., $13$. (**B**) Lollipop plot showing the distribution of significant GO-MF terms associated with DEIRGs. The $\mathrm{x}$ and $\mathrm{y}$ axes represents the MF terms and gene count. The term “signaling receptor activator activity” has highest number of genes present in it, i.e., $41$. The term “protein tyrosine kinase activator activity” has lowest number of genes present in it, i.e., $4$. (**C**) Circos plot representing significant GO-CC terms associated with DEIRGs. Outer boundary of the circle consists of $6$ CC terms (on the left) linked with the DEIRGs present in them (on the right). Each gene and pathway are denoted by unique color strips with an undirected edge showing the corresponding associations. (**D**) Circular barplot showing the distribution of $8$ significant pathways associated with DEIRGs. The color and sizes of bars depend on the $\mathrm{q}-\mathrm{value}$ and gene count, respectively.

**Figure 2:**
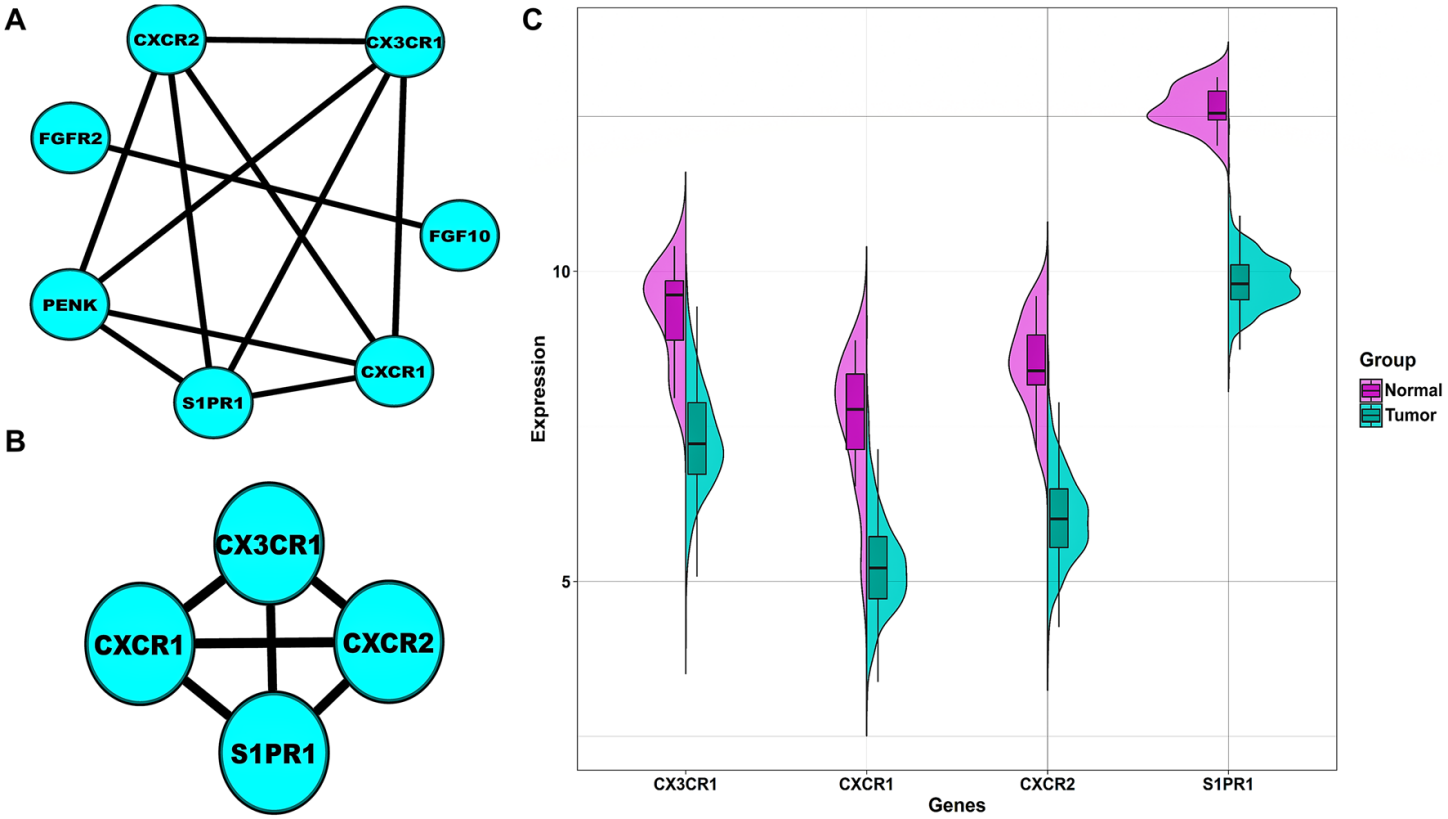
(**A**) Unweighted and undirected PPIN of significantly enriched DEIRGs comprising $7$ nodes and $30$ edges. (**B**) Top-scoring PPIN cluster comprising a total of $4$ nodes and $30$ edges. Cyan-colored nodes signify downregulated expression status of DEIRGs. (**C**) Split violin plot displaying the expression intensity distribution of $4$ hub DEIRGs. The normal and tumor samples are represented by magenta and sea green colors. The top and bottom of the boxes inside the splitted violin depicts the $75$th and $25$th percentile of the distribution, respectively. The horizontal lines within the boxes signifies the median values. Axis endpoints are labelled by the minimum and maximum values, respectively.

We retrieved $1793$ IRGs from the ImmPort database and $129$ DEIRGs overlapped between our DEGs and IRGs. Venn plot representing the number of DEGs, IRGs, and DEIRGs is shown in Fig. S1B. A total of $110$ DEIRGs out of $129$ were actively involved in $52$ significantly enriched GO terms (i.e., $14$ BP terms $+$ $32$ MF terms $+$ $6$ CC terms). Treemap illustrating the significant BP terms with respect to the number of DEIRGs is shown in Fig. 1A. The number of DEIRGs involved in BP terms ranged from $13$ to $27$, respectively. A lollipop plot showing the significant MF terms as lollipops and their corresponding DEIRG count is shown in Fig. 1B. The number of genes in these MF terms ranged from $4$ to $41$. Circos plot showing the association of DEIRGs and significant CC terms is shown in Fig. 1C. The number of DEIRGs in these CC terms ranged from $7$ to $14$. Moreover, $52$ DEIRGs actively participated in $8$ significantly enriched pathways. Circular barplot showing the significant pathways with respect to their gene count is shown in Fig. 1D. A total of $9$ DEIRGs overlapped among all the significantly enriched pathways and GO terms and is shown by the Venn plot in Fig. S2. Amongst these DEIRGs, only $7$ took part in the PPIN corresponding to the aforementioned threshold (i.e., $\mathrm{confidence score }>0.9$). Figure 2A shows the PPIN comprising $7$ DEIRGs linked by $11$ edges. The top-scoring PPIN cluster identified using MCODE comprised $4$ hub DEIRGs linked by $6$ edges as shown in Fig. 2B, respectively. A split violin plot showing the distribution of expression of $4$ hub DEIRGs is shown in Fig. 2C. *S1PR1* had a considerably higher expression level than the other three hub DEIRGs, as evidenced from the violin plot.

### miRNA-mRNA-TF regulatory network analysis and ROC curve validation

**Figure 3:**
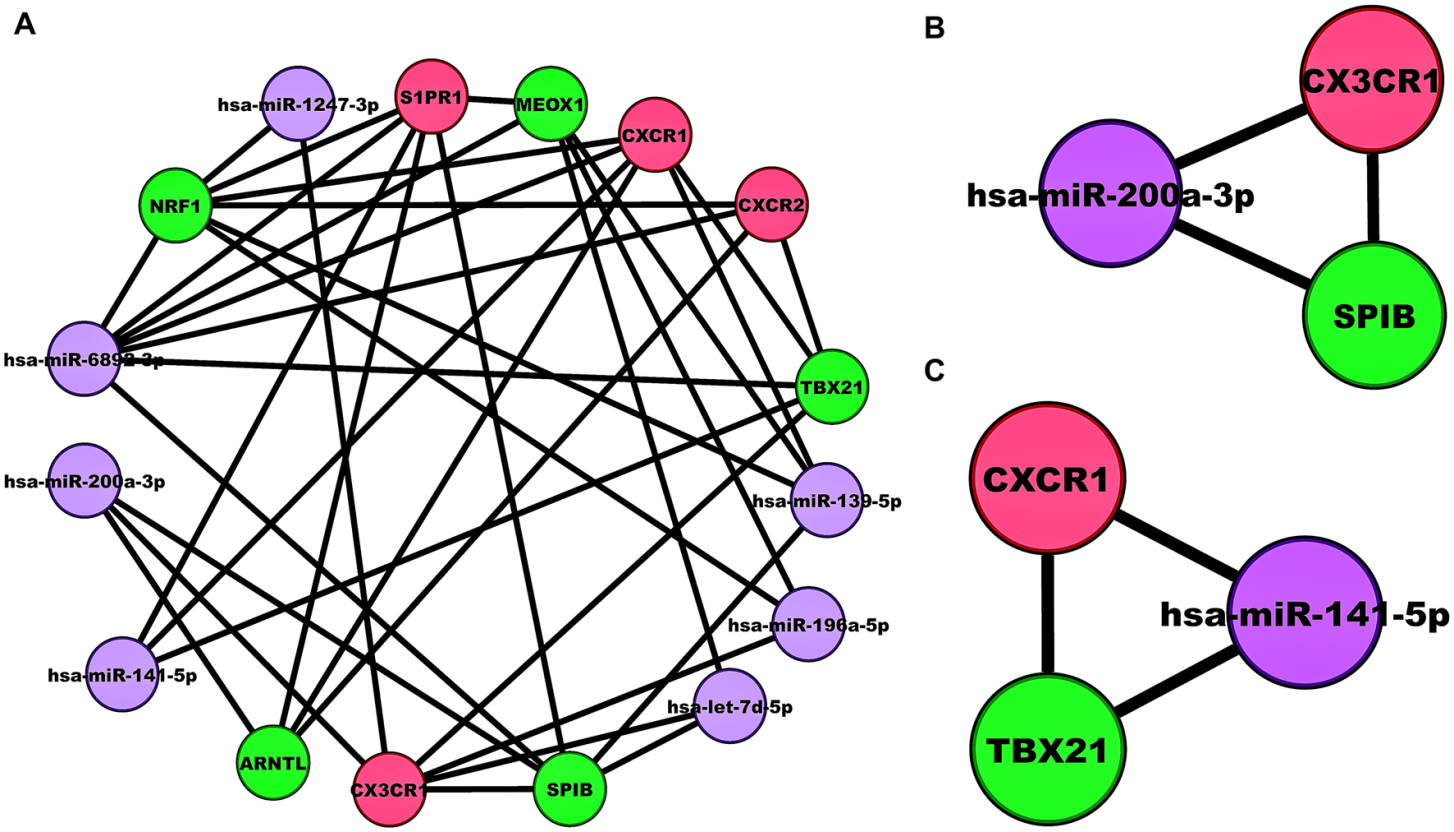
(**A**) Unweighted LUAD-specific $3$-node miRNA FFL comprising $30$ nodes and $30$ edges. (**B**) miR-200 family-associated highest-order subnetwork motif comprising one miRNA (hsa-miR-200a-3p), one TF (*SPIB*), and one hub DEIRG (*CX3CR1*). (**C**) miR-200 family-associated second highest-order subnetwork motif comprising one miRNA (hsa-miR-141-5p), one TF (*TBX21*), and one hub DEIRG (*CXCR1*). The green, red, and magenta-colored nodes represents the TFs, hub DEIRGs, and miRNAs, respectively.

**Figure 4:**
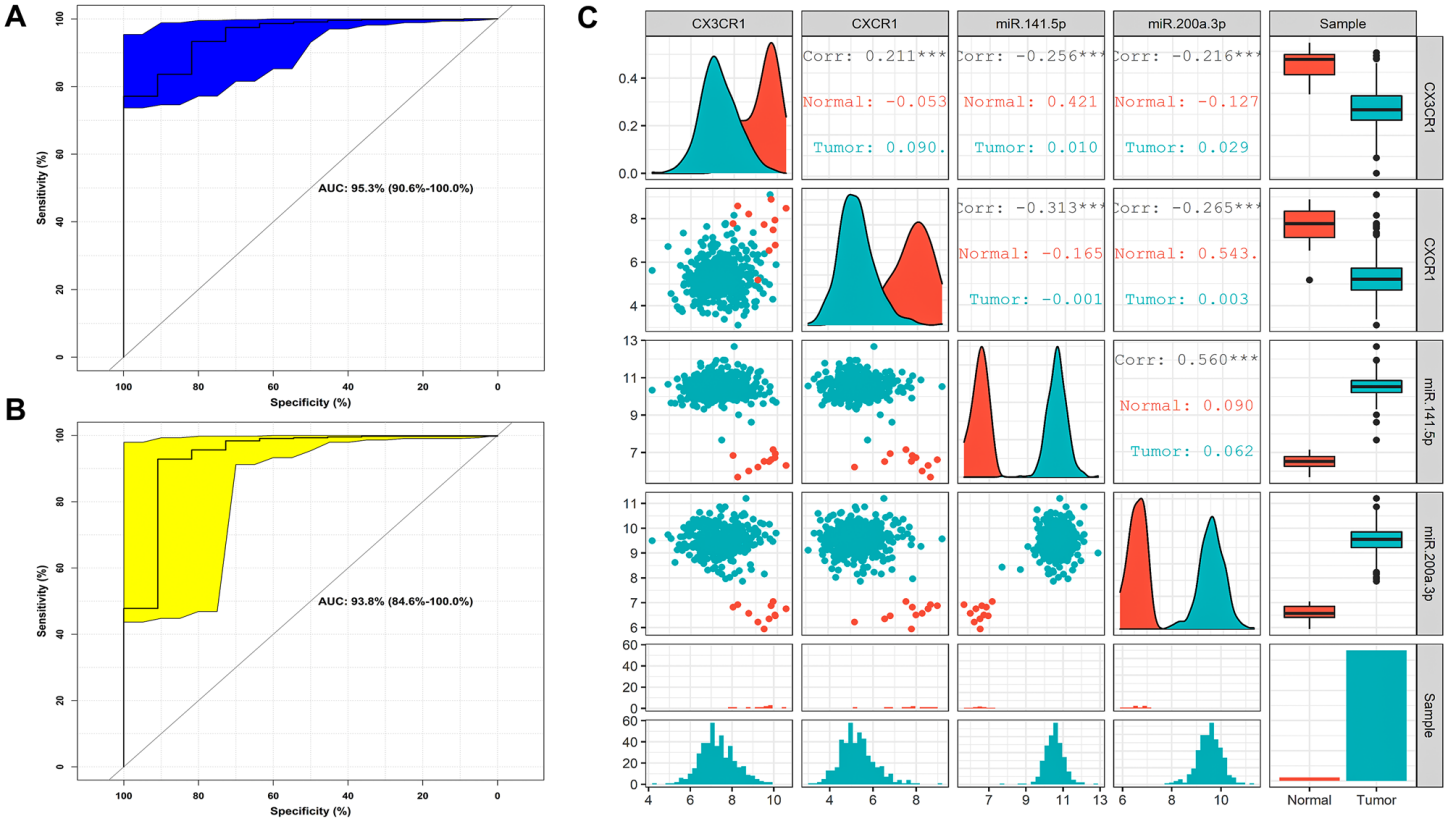
ROC curve analyses of (**A**) *CX3CR1* and (**B**) *CXCR1*. (**C**) Pairwise scatter plot of miR-200 associated hub items, i.e., *CX3CR1*, *CXCR1*, hsa-miR-200a-3p, and hsa-miR-141-5p. The upper triangular section represents the Spearman’s correlation coefficients between these hub items. While the lower triangular section represents the scatterplot and histogram distribution between these hub items. The diagonal consists of kernel densities for each hub item. Significant levels at $0.05$, $0.01$, and $0.001$ are represented by *, **, and ***, respectively.

Our $3$-node miRNA FFL regulatory network included a total of $16$ nodes and $37$ edges as shown in Fig. 3A. Amongst all the edges, $12$, $10$, and $15$ edges belonged to TF-mRNA, miRNA-mRNA, and miRNA-TF pairs, respectively. Amongst all the nodes, $7$, $4$, and $5$ nodes belonged to miRNAs, mRNAs, and TFs, respectively. The degree values of miRNAs, mRNAs, and TFs ranged from $1$ to $7$, $1$ to $6$, and $1$ to $7$, respectively. The average degrees of miRNAs, mRNAs, and TFs were $3.125$, $2.75$, and $3.375$, respectively. We observed two highest-order subnetwork motifs with respect to the miR-200 family. The first subnetwork motif included one miRNA (miR-200a-3p), one mRNA (*CX3CR1*), and one TF (*SPIB*) as shown in Fig. 3B. Whereas the second one included one miRNA (miR-141-5p), one mRNA (*CXCR1*), and one TF (*TBX21*) as shown in Fig. 3C. The ROC curve analysis of *CX3CR1* and *CXCR1* is shown in Figs. 4A-B, respectively. The $95\%\mathrm{CI}$ and AUC of *CX3CR1* were $0.906-1$ and $0.953$, and those of *CXCR1* were $0.846-1$ and $0.938$, respectively. Pairwise scatterplot matrices exhibiting association between *CX3CR1*, *CXCR1*, miR-200a-3p, and miR-141-5p is shown in Fig. 4C. Both the miRNAs (i.e., miR-141-5p and miR-200a-3p) had the highest correlation value. i.e., $0.560$ as evidenced from the plot.

### Tumor immune infiltration analysis

**Figure 5:**
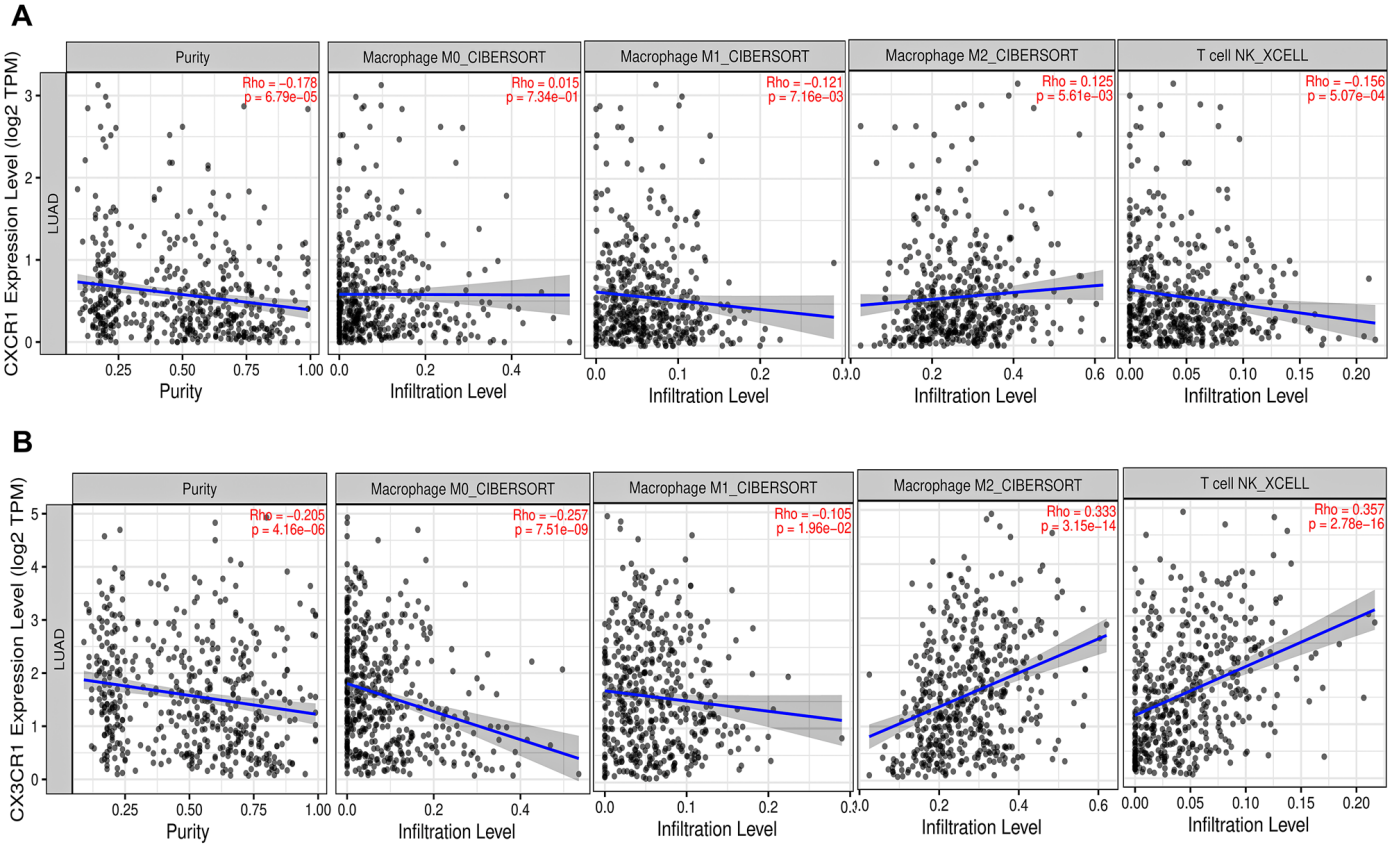
Scatterplots showing significant correlations of (**A**) CXCR1 and (**B**) CX3CR1 mRNA expression with M0, M1, M2, NKT infiltrating levels across the TCGA-LUAD cohort. mRNA expression levels against tumor purity are demonstrated on the left panel. Spearman’s correlation and estimated statistical significance are displayed for each scatter plot.

*CXCR1* and *CX3CR1* expression levels were significantly correlated with tumor purity and the infiltrating levels of macrophages and NKT in LUAD. M0 macrophages were significantly correlated with *CX3CR1* ($\mathrm{r}=-0.257$, $p=7.51\times {10}^{-9}$) whereas nonsignificantly correlated with *CXCR1* ($\mathrm{r}=0.015$, $p=7.34\times {10}^{-1}$) as shown in Fig. 5. Infiltrating levels of M1 macrophages had significant correlation with the expression levels of both *CX3CR1* ($\mathrm{r}=-0.105$, $p=1.96\times {10}^{-2}$) and *CXCR1* ($\mathrm{r}=-0.121$, $p=7.16\times {10}^{-3}$) (Fig. 5). But, infiltration levels of M2 polarized macrophages were significantly associated with the expression levels of both *CX3CR1* ($\mathrm{r}=0.333$, $p=3.15\times {10}^{-14}$) and *CXCR1* ($\mathrm{r}=0.125$, $p=5.61\times {10}^{-3}$) (Fig. 5). Similarly, *CXCR1* ($\mathrm{r}=-0.156$, $p=5.07\times {10}^{-4}$) had significant negative while *CX3CR1* ($\mathrm{r}=0.357$, $p=2.78\times {10}^{-16}$) had significant positive correlation with NKT infiltrating levels as shown in Fig. 5. In addition, both these genes were significantly negatively correlated with tumor purity in LUAD. These results indicate the clear role of both *CX3CR1* and *CXCR1* in immune responses in LUAD TME as suggested by their significant positive correlation with M2 macrophage infiltration and significant correlations with NKT infiltrations.

### Validation of miR-200 family-associated hub DEIRGs using HPA and GEO

**Figure 6:**
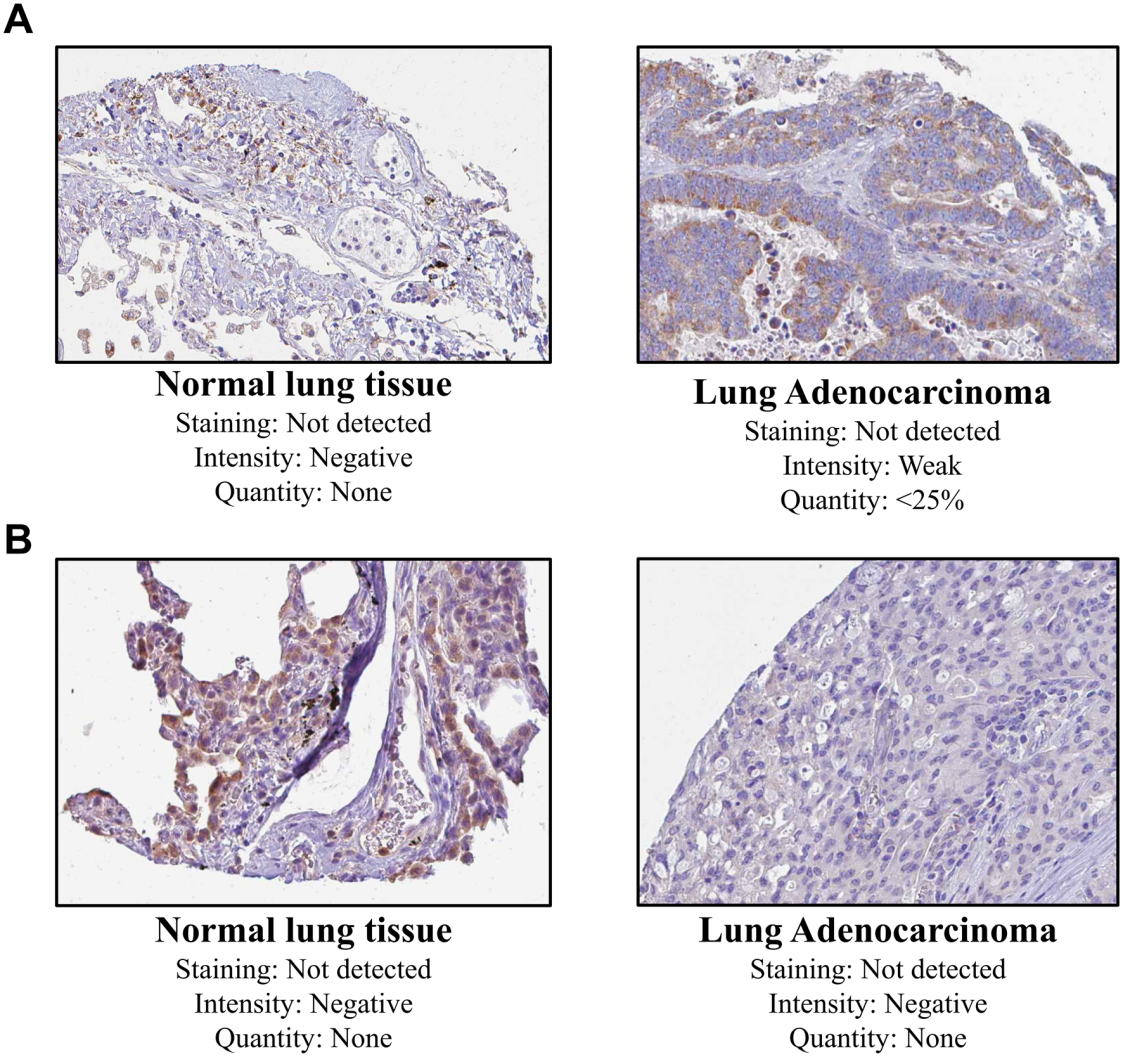
Representative IHC images of (**A**) CX3CR1 and (**B**) CXCR1 across normal and LUAD tissues via HPA database.

In HPA database, the protein expression levels of both CX3CR1 and CXCR1 in the LUAD sample tissues were distinct from the corresponding normal lung tissue samples (Fig. 6). GSE116959 ($11$ healthy control + $57$ tumor tissues) and GSE43458 ($30$ healthy control + $80$ tumor tissues) mRNA expression profiles were chosen in accordance with the abovementioned exclusion/inclusion criteria. Both *CXCR1* and *CX3CR1* were present in the DEGs list, thus affirming their validation in external GEO datasets. Figure S3 depicts box-and-whisker plots of the relative expression distributions of *CXCR1* and *CX3CR1* across LUAD patient samples compared to healthy normals. As observed, both genes' mRNA expression levels were significantly downregulated in tumor samples compared to healthy normals.

### Prognostic assessment of miR-200 family-associated hub items

**Figure 7:**
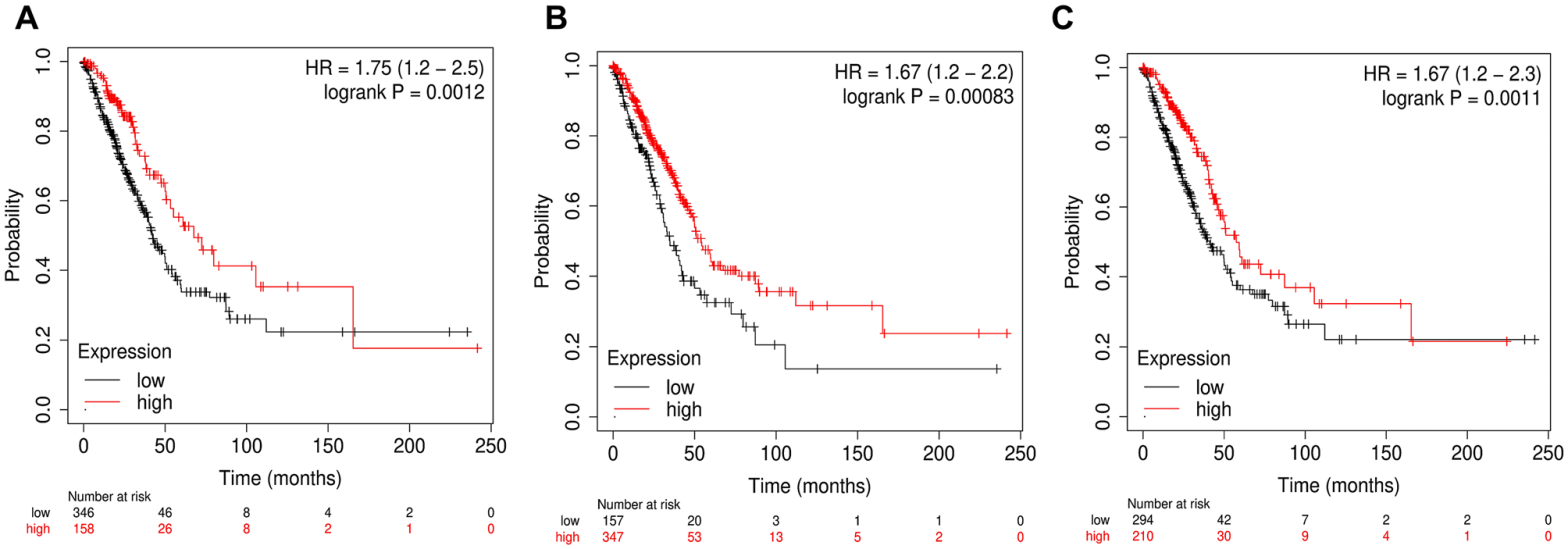
KM plots showing OS of (**A**) *CX3CR1*, (**B**) miR-200a-3p, and (**C**) *SPIB*. The black and red colored lines corresponds to LUAD samples with lower and higher expression levels, respectively. The lower expression of all these subnetwork motif items correlates with lower OS in LUAD patients. All these motif items were highly significant ($\mathrm{log rank } p < 0.05$).

The corresponding KM plots shown in Fig. 7A–C depicted that lower expression levels of *CX3CR1* ($\mathrm{HR}=1.75$; $95\%\mathrm{CI}=1.2-2.5$; $p<0.05$), miR-200a-3p ($\mathrm{HR}=1.67$; $95\%\mathrm{CI}=1.2-2.2$; $p<0.05$), and *SPIB* ($\mathrm{HR}=1.67$; $95\%\mathrm{CI}=1.2-2.3$; $p<0.05$) worsened the OS in $513$ LUAD patients. The low and high expression cohort median survival times of each item is detailed in table S1, respectively.

### Expression of fractalkine receptor CX3CR1 and its ligand CX3CL1 in overexpression model of miR-200a-3p cultured in macrophage-conditioned media

To study the critical role of miR-200a-3p regulated identified hub gene *CX3CR1* (fractalkine receptor) in TME, scrambled or miR-200a-3p transfected LUAD cell lines A549 and H1299 were grown in M0/M1/M2 macrophage-conditioned media. The expression of *CX3CR1* and its only known ligand, the chemokine *CX3CL1* (a.k.a Fractalkine), was quantified using qRT-PCR in M0/M1/M2 macrophages as well as in scrambled/miR-200a-3p transfected A549 and H1299 cells treated with macrophage-conditioned media.

**Figure 8:**
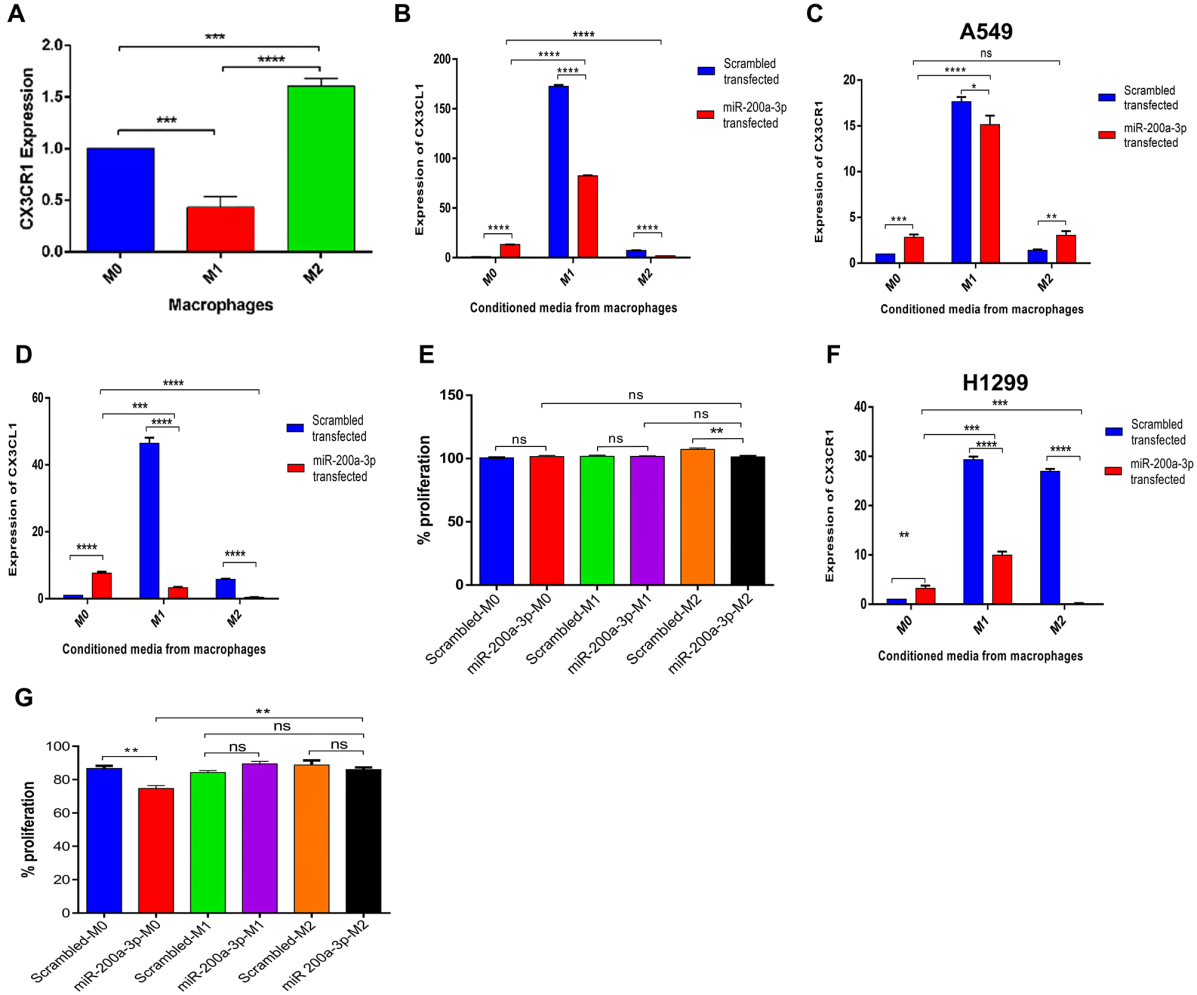
(**A**) Relative fold change in expression of *CX3CR1* in M0 (unstimulated), M1 (LPS stimulated) and M2 (IL4 stimulated) macrophages. Relative fold change in the expression of (**B**) *CX3CL1* and (**C**) *CX3CR1* upon transfection of scrambled/ miR-200a-3p in A549 cells indirectly co-cultured in M0/M1/M2 macrophage conditioned medium as determined by qRT-PCR. (**D**) Percentage proliferation of scrambled/ miR-200a-3p transfected A549 cells when indirectly co-cultured in M0/M1/M2 macrophage conditioned medium, as determined by MTT assay. Relative fold change in the expression of (**E**) *CX3CL1* and (**F**) *CX3CR1* upon transfection of scrambled/ miR-200a-3p in H1299 cells indirectly co-cultured in M0/M1/M2 macrophage conditioned medium as determined by qRT-PCR. (**G**) Percentage proliferation of scrambled/ miR-200a-3p transfected H1299 cells when indirectly co-cultured in M0/M1/M2 macrophage conditioned medium, as determined by MTT assay. $\upbeta$-actin was used as an endogenous control. ns- Nonsignificant, *p < 0.05, **p < 0.01, ***p < 0.001.

The expression of *CX3CL1* was very low in M0 subset macrophages and even further lower in M1 macrophages while it was undetectable in case of M2 polarized macrophages (data not shown). The *CX3CR1* expression in M1 polarized macrophages was significantly decreased as compared to M0 and M2 subsets. While it was significantly higher in M2 polarized macrophages than both M0 and M1 macrophages subsets (Fig. 8A).

M0 macrophages are the undifferentiated subset of macrophages that can differentiate into a specific polarized state of macrophages like M1 which are pro-inflammatory or M2 which are anti-inflammatory. In the presence of M0 macrophage-conditioned media, miR-200a-3p transfected A549 and H1299 cells showed a highly significant increase in the expression of *CX3CL1* (Fig. 8B,E) in comparison to scrambled transfected cells. Similarly, a significant increase in the relative expression of fractalkine receptor *CX3CR1* was also quantified in both miR-200a-3p transfected A549 and H1299 cells cultured in M0-conditioned media (Fig. 8C,F). This indicated a clear role of miR-200-3p in increasing *CX3CL1* and *CX3CR1* expression in cancer cells. The expression of *CX3CL1* was significantly lower in both miR-200a-3p transfected A549 and H1299 cells when grown in the presence of M1 and M2 conditioned media as compared to scrambled transfected corresponding control cells. However, the expression level of *CX3CL1* was much higher when cells were grown in M1 macrophage-conditioned media than when the cells were grown in M2 macrophage-conditioned media (Fig. 8B,E). This indicates that *CX3CL1* (Fractalkine) expression is much higher in pro-inflammatory TME, suggesting the cancer-eradicating role of *CX3CL1*-*CX3CR1* axis, possibly by inducing apoptosis in cancer or immune targeting of cancer cells leading to anti-cancer processes. However, the significant decrease in *CX3CL1* after miR-200a-3p transfection in both pro-inflammatory and anti-inflammatory TMEs suggests the association of miR-200a with decreased immune effector cell infiltration and decreased tumor-suppressive activity, which may also result in higher metastasis. Although, *CX3CL1* additionally has tumor-suppressive activity as *CX3CL1* overexpression has been shown to increase the chemotactic efficiency and infiltration of immune effector cell, resulting in an improved prognosis.

The expression of the fractalkine receptor *CX3CR1* also increased in both miR-200a-3p transfected A549 and H1299 cell lines when cultured in M0-conditioned media (Fig. 8C,F). The expression of *CX3CR1* decreased significantly in miR-200a transfected A549 cell line, and a highly significant decrease was observed in miR-200a transfected H1299 cell line when cultured in M1 macrophage-conditioned medium indicating a chemotactic role of *CX3CR1* here in pro-inflammatory TME. Surprisingly in M2 conditioned medium, miR-200a-3p transfected A549 cell line showed a highly significant increase in *CX3CR1* receptor as compared to scrambled transfected cells. However, in H1299, there was a significant reduction in *CX3CR1* expression in miR-200a-3p transfected cells when grown in M2-conditioned media. Increased *CX3CR1* expression is associated with increased M2 macrophage infiltration and increased *CX3CR1* expression has also been associated with metastasis-initiating cells.

### Tumor cell proliferation upon miR-200a-3p overexpression on culturing with macrophage-conditioned medium

To further assess if the expression of *CX3CL1*-*CX3CR1* and miR-200a-3p was correlated with the proliferation ability of A549 and H1299 cell line when grown in the presence of M0, M1 and M2 macrophage-conditioned media, MTT assay was performed. There was a nonsignificant difference in the proliferation ability of scrambled or miR-200a-3p transfected A549 cells when cultured in M0 and M1 macrophage-conditioned media. However, there was a significant decrease in proliferation of miR-200a transfected A549 cells compared to scrambled transfected when cultured in M2 macrophage-conditioned media indicating that miR-200a overexpression leads to decreased proliferation of cancer cells in TME. However, this difference was insignificant when cells were grown in either M0, M1 or M2 macrophage-conditioned media (Fig. 8D).

In H1299 cell line there was no difference in the proliferation ability of miR-200a transfected cells when cultured in M1 or M2-conditioned medium (Fig. 8G). The proliferation ability of H1299 cell line decreased when cultured in M0 macrophage condition medium. The proliferation assay in both A549 and H1299 cells indicated that the LUAD cell proliferation ability was not correlated with the expression of *CX3CL1* or *CX3CR1*. This is indicative of the dual role of *CX3CL1-CX3CR1* axis in TME.

## Discussion

Carcinogenesis of LUAD is among one of the most lethal malignancies, which is a complex and multistage process regulated by many genes and miRNAs^34^. Genetic regulations involved in TME including tumor inflammation and immunity play a critical decisive role at various stages of tumor development^6^. Our study assumes TME as a key player in tumor growth and metastasis which may pave the way for identifying therapeutic and prognostic targets for early detection and treatment of LUAD for increased OS of LC patients. The present study identified DEMs and DEGs in the TCGA-LUAD cohort followed by $129$ DEIRGs detection. Our study specifically targeted regulatory network motifs associated with miR-200a-3p and miR-141-5p which were our DEMs of interest in LUAD. The pathway and GO term analysis results showed that the BP were enriched in cell chemotaxis, second messenger mediated signaling, etc., affirming the critical role of IRGs in immune response in TME. In this study, we further validated the role of miR-200a-3p in TME in vivo using miR-200a-3p transfected A549 (wild type p53 LUAD) and H1299 (null p53 LUAD) cells cultured in M0, M1 (pro-inflammatory) and M2 (anti-inflammatory) macrophage-conditioned medium.

The first highest order miR-200 family-associated subnetwork motif comprised miR-200a-3p, *CX3CR1* and *SPIB*. miR-200 family has been reported to function as an oncogene or tumor-suppressor in several carcinogenesis, but its crucial biological importance and functions in NSCLC are subtle. The miR-200 family is known to target *ZEB1* and *ZEB2*, which maintains the epithelial phenotype of cells mediated by transcriptional repression of E-cadherin. *ZEB1* and *ZEB2* can induce EMT leading to cancer cell migration, invasion, and metastasis. ${\varvec{\upbeta}}$-catenin mRNA is a direct target of miR-200a, and it can suppress the β-catenin/Wnt signaling pathway which is commonly involved in cancer^35^. Moreover, miR-200a expression was negatively associated with cyclin D1 and $\upbeta$-catenin in human meningioma tumor tissues^36^. It was suggested that miR-200a-3p was able to induce EMT in NSCLC and serve as a tumor promoter.

*CX3CL1* is the only known member of chemokine CX3C family and *CX3CR1* is the only receptor of CX3CL1 (Fractalkine). *CXCL1* (membrane-bound/secreted) can potentially regulate tumor-related inflammatory response*. CX3CR1* (the fractalkine receptor) is expressed in NKT cells, ${\mathrm{CD}8}^{+}$ T cells, DCs, and monocytes. The *CX3CL1*–*CX3CR1* axis is reported to be upregulated in LC, colon cancer, breast cancer, gastric cancer, prostate cancer, and other malignancies. Our study emphasizes the different pattern of expression and the role of *CX3CL1*-*CX3CR1* axis in pro-inflammatory (M1 macrophages) and anti-inflammatory (M2 macrophages) TME. Indeed, the high expression of *CX3CR1* in M2 macrophages obtained in our study corroborated with other studies considering *CX3CR1*-expressing ${\mathrm{CD}68}^{+}{\mathrm{CD}206}^{+}$ cells in the lungs M2-macrophages^37^. While M1 macrophages are shown to increase the expression of *CX3CL1*-*CX3CR1* axis supporting the theory that *CX3CL1* can attract immune effector cells to the tumor location site and exert an anti-tumor immune effect. However, the downregulation of both *CX3CL1* and *CX3CR1* when cultured in M2 macrophage-conditioned medium supports the dual functional role of *CX3CL1*. This discrepancy may be attributed to the dual role of *CX3CL1* acting both as a chemoattractant for leukocytes as well as an adhesion molecule for the tumor cells.

The clinical role of *CX3CL1*-*CX3CR1* signaling has been reported to be contradictory and it may exert both pro-tumor and anti-tumor effects based on tumor tissue and histological grade of tumor^38,39^. *CX3CL1* may also promote the adhesion of *CX3CR1*-positive tumor cells to target organs, thus causing the migration of tumor cells and promoting tumorigenesis^40^. miR-200a expression is downregulated in NSCLC, thus acting as tumor suppressor. Our study observed that miR-200a-3p upregulates the expression of *CX3CR1*, indicating the critical role of miR-200a-3p as a regulator of metastasis to distant organs and EMT. And hence the results can be corroborated with the study suggesting a correlation of miR-200a with advanced stages of NSCLC and low survival. Thus the upregulation of *CX3CR1* in our study may be correlated with the poor prognosis and role of miR-200a in EMT. Our study supports the tumorigenic and metastatic role of miR-200a via modulating the expression of *CX3CR1* in TME.

In contrast to this, the study shows the reduction in *CX3CR1* expression on transfection of miR-200a-3p transfection in H1299. This indicates the role of p53-mediated effect of miR-200a in regulating *CX3CR1* expression. Several studies pointed out the essential role of Src/FAK signaling pathway in *CX3CL1*-*CX3CR1* axis-mediated migration and invasion of LC and breast cancer cells^41,42^. Further study will be needed to elucidate the most probable involvement of Src/FAK pathway regulation via miR-200a in LC. Interestingly, these cells were also reported to have a higher *CX3CR1* mRNA expression. Emerging studies also suggest *CX3CR1* as a marker of stem-like tumor cells and cells with relatively higher *CX3CR1* expression show transcriptomic profiles enriched in pathways regulating pluripotency. In murine models, these cells resist chemotherapy and metastasis-initiating behavior^43^.

Expression of *CX3CR1* is heterogenous even between cancer subtypes is associated with histological grade and stage-dependent progression of various malignancies^38,44^. Tumor cells which express *CX3CL1* can induce the invasion and metastasis of *CX3CR1*-positive tumor cells^45,46^. It was shown earlier that the mRNA and protein expression of CX3CL1 and CX3CR1 was significantly high in primary LC and secondary bone metastasis. Serum levels of both were positively correlated to the LC progression. Thus, it was suggested that the *CX3CL1*–*CX3CR1* axis is associated with LC growth and metastasis^47^. Similarly, increased *CX3CR1* expression was correlated with bone metastasis in prostate cancer, similarly in breast cancer, expression of *CX3CR1* predicted the occurrence of BM^40,48^. Fractalkine and *CX3CR1* are recognizably capable of facilitating the adhesion and extravasation of *CX3CR1*-expressing circulating tumor cells into skeleton and soft-tissue organs and facilitating colonization and progression of tumor into secondary organs^49^. CX3CL1 induced cell migration in human osteosarcoma cells via upregulating *ICAM-1* expression mediated by CX3CR1/PI3K/Akt/NF-κBpathway^44^. FKN/CX3CR1 activated JAK/STAT signaling in PDAC, which could further regulate cell growth and EMT. Inhibition of *CX3CR1* was reported to inhibit the cancer cell survival and increase sensitivity towards chemotherapy in NSCLC^50^. *CX3CR1* activates pro-survival signaling pathways in normal and cancer cells thus promoting cell viability^51^. It is known to act via Wnt and notch signaling pathways^52,53^. It may be predicted that miR-200a targets Wnt and notch signaling possibly via *CX3CR1* axis to target metastasis in LUAD.

The cell proliferation ability of miR-200a-3p transfected A549 and H1299 cells did not correlate with the corresponding *CX3CL1*-*CX3CR1* axis expression. This could be because the cytokines are mainly associated with EMT and metastasis processes and do not significantly affect the cancer cell apoptosis in TME. A recent report in human pancreatic cancer cells indicated that *CX3CL1* protects against apoptosis. Pancreatic cancer cell lines upregulated the expression of anti-apoptotic molecules like *BCL-2* and BCL-xl in response to exogenous *CX3CL1*; while *CX3CL1* stimulation caused the decrease in the expression of the pro-apoptotic caspase 3. The mechanisms found depended on AKT phosphorylation^54^.

The lymphocyte-restricted protein, Spi-B, is ectopically expressed in LCs, and its increased expression level is correlated with poor prognosis in human LC. Spi-B was the hub TF involved in the regulatory network of miR-200a and *CX3CR1*. Earlier studies show that Spi-B was expressed in invasive cancer cells in human primary LC tissues. Vimentin was co-expressed with Spi-B whereas E-cadherin was repressed in LC. Spi-B-expression was also associated with lymphatic metastasis, short OS and tumor grade. Spi-B also downregulated claudin-2, disrupting intercellular junctions and enhancing invasiveness in LC cells^55^. *SPIB* activation significantly increased anoikis resistance from loss of attachment‐induced autophagy^56,57^.

In our study, another FFL subnetwork motif of miR-200 family-mediated regulation included miR-141, *CXCR1* and *TBX21*. Role of miR-141-5p is largely unclear in NSCLC. Lower expression of miR-141-5p was associated with poor patient survival as an independent risk factor, lymph node metastasis, and advanced TNM stage^58^. In another study, higher expression of miR-141 in serum was associated with shorter OS in LUAD patients^59^. miR-141 expression also improved the secretion of *VEGFA*. *VEGFA* is associated with CAFs and tumor invasion in LUAD models in murine LUADs. This is mediated by downregulation of *KLF6*^5,60^ and induced neoangiogenesis. miR-141 regulates the expression of *PHLPP1* and *PHLPP2*, antagonists of PI3K/AKT signaling and promotes the proliferation of NSCLC cells^61^. This further indicates the critical role of miR-141-5p circuitry in regulating LUAD progression. Till now, no study has reported the role of miR-141 in the regulation of IRGs in TME of NSCLC which may enhance the invasion and metastasis. Expression of *CXCL8* chemokine which has angiogenic and pro-inflammatory activity is also known to affect tumor cells, inducing the proliferation of LC cells through *CXCR1*^62^.The *CXCL8* (secreted by tumor cells)-*CXCR1/2* (in TME) axis may also regulate CSC proliferation and self-renewal thus play a critical role in LC progression and metastasis in TME^63^.

*CXCR1* can interact with both the ligands, *CXCL6* and *CXCL8*. *CXCL6* is known to be upregulated in LC^64^. miR-141 was found to be downregulated in the *CXCL6* treated A549 cells^65^. *CXCR1* is expressed on monocytes, some NK cells, granulocytes, and mast cells^66^. *CXCR1* alone is associated with *CXCL8*-mediated chemotaxis^67^. *TBX21*, aTh1 cell-specific TF, was earlier identified as an independent predictive factor in LUAD. *TBX21* was correlated with cancer stemness mediated by the *TBX21*-*IL-4* pathway in LUAD patients^68^. *TBX21* was associated with poor prognosis in LUAD.

There are controversial studies reported with respect to the high or low expression of miR-200 family in LUAD which significantly reduces the median OS in LUAD patients. Our study reported the survival plot of the LUAD patients with relation to miR-200a, *CX3CR1* and Spi-B. All of them significantly (${p }< 0.05$) affected the OS in the LC patients with the median OS in lower expression cohort of miR-200a, *CX3C1* and Spi-B being $34.87$ months, $42.17$ months and $41.17$ months respectively (Fig. 7). The controversies reported in different reports may be due to the source of miR-200 family used in expression studies, i.e. either tissue or serum. The expression of *CX3CR1* and *CXCR1* was further correlated with the IHC images provided in the HPA database (Fig. 6).

The tumor immune infiltration analysis was done using TIMER. Our study reported that *CXCR1* and *CX3CR1* expressions were significantly negatively correlated with tumor purity whereas significantly positively correlated with infiltrating levels of macrophage in LUAD, especially M2 macrophages. *CXCR1* had a significant negative correlation while *CX3CR1* had a significant positive correlation with infiltrating levels of NKT in LUAD.

As cancer is designated as a chronic inflammatory disease, thus tumor progression is dependent on inflammatory microenvironment. *CX3CR1* facilitates macrophage survival, leading to enhanced angiogenesis and metastasis of tumor cells. Deficiency of *CX3CR1* diminished the extent of macrophages infiltrating the metastatic foci by inducing macrophage apoptosis^69^. Thus, our studies indicate that *CX3CR1* regulated by miR-200a further aggravates the metastasis in LUAD mediated by inflammatory macrophages as indicated by positive correlation obtained in our studies. Few studies also indicated the possible role of *CX3CR1*-mediated angiogenic switch regulating malignant transition where macrophage may play a pivotal role in vascular remodelling in different tumor models^69–71^. However, this needs to be further correlated to TME studies. *CX3CR1* is also expressed on T cell and NKT cell subsets. NKT, also called CD1d-restricted T cells, are a heterogeneous group of T cells that also share the properties of NK cells. TH_1_-like NKT cells may induce an antitumor response whereas, TH_2_- and T_reg_-like NKT cell subsets may mediate tumor progression and immune escape. Overstimulated NKT cells may skew toward TH_2_-/T_reg_-like subsets, thus mediating immune escape and tumor progression^72^. No studies had been done to reveal the association of *CX3CR1* and NKT in TME.

Increased tumor angiogenesis and shorter median OS of LC are associated with the expression of *IL8* mRNA, which is known to act through *CXCR1/2*, in the lung TME induced by infiltrating macrophages via the NFKB pathways^73^. One other study correlated *CXCR1* expression intensity with tumor infiltration by macrophages and total number of tumor-infiltrating immune cell^74^, however the exact role of *CXCR1* in macrophage or NKT cells mediated tumor progression has not been yet revealed. Our study could improve our comprehension in miR-200 family regulated role of IRGs in TME in LUAD.

## Conclusion

TME is a complex player in the progression and metastasis of tumor. The study emphasizes the miR-200 family-regulated IRGs as key players in LC, leading to aggravated metastasis and reduced OS in LUAD patients. These IRGs could also affect tumor-infiltrating cell population in LUAD which was correlated with the possible role of these immune effector cells in tumor progression, invasion, and metastasis. The study provided insight into the role of miR-200a- 3p mediated regulation of *CX3CL1*-*CX3CR1* axis, which may be associated with progression of LC. Thus, targeting miR-200a-*CX3CR1*-SPiB and miR-141-*CXCR1*-*TBX21* axes may provide a potential prognostic as well as therapeutic target for efficient management of LUAD-related metastasis and poor survival.

### Supplementary Information

Supplementary Information.

## Data Availability

The data used in our study was downloaded from https://xenabrowser.net/datapages/?cohort=GDC%20TCGA%20Lung%20Adenocarcinoma%20(LUAD)&removeHub=https%3A%2F%2Fxena.treehouse.gi.ucsc.edu%3A443.

## Abbreviations

NSCLC: Non-small cell lung cancer
LUAD: Lung adenocarcinoma
LC: Lung cancer
OS: Overall survival
miRNA: MicroRNA
mRNA: Messenger RNA
EMT: Epithelial-mesenchymal transition
TME: Tumor microenvironment
DEGs: Differentially expressed genes
DEMs: Differentially expressed miRNAs
TCGA: The cancer genome atlas
DEIRGs: Differentially expressed immune-related genes
FFL: Feed-forward loop
ECM: Extracellular matrix
CAFs: Cancer-associated fibroblasts
TFs: Transcription factors
KM: Kaplan–Meier
TIMER: Tumor immune estimation resource
HPA: Human protein atlas
HTseq: High-throughput sequence
HGNC: HUGO gene nomenclature committee
BH: Benjamini–Hochberg
PPIN: Protein–protein interaction network
DEA: Differential expression analysis
ImmPort: Immunology database and analysis portal system
IRGs: Immune-related genes
GO: Gene ontology
ROC: Receiver operating characteristics
AUC: Area under curve
LPS: Lipopolysaccharide
RPMI: Roswell park memorial institute medium
IL-4: Interleukin 4
rpm: Revolutions per minute
qRT-PCR: Quantitative real-time PCR
DMSO: Dimethyl sulfoxide
SEM: Standard error of the mean
BP: Biological process
MF: Molecular function
CC: Cellular compartment
CI: Confidence interval
NKT: Natural killer T
HR: Hazard ratio
ZEB1: Zinc finger E-box binding homeobox 1
ZEB2: Zinc finger E-box binding homeobox 2
VEGFA: Vascular endothelial growth factor A
CSC: Cancer stem cell
SFTPA1: Surfactant protein A1
SFTPA2: Surfactant protein A2
GDF10: Growth differentiation factor 10
SFTPD: Surfactant protein D
S1PR1: Sphingosine-1-phosphate receptor 1
CX3CR1: C-X3-C motif chemokine receptor 1
SPIB: Spi-B transcription factor
CXCR1: C-X-C motif chemokine receptor 1
TBX21: T-Box transcription factor 21
CX3CL1: C-X3-C motif chemokine ligand 1
ICAM-1: Intercellular adhesion molecule-1;
UCSC: University of California, Santa Cruz
GDC: Genomic data commons
DC: Dendritic cell
ncRNA: Non-coding RNA
NCCS: National centre for cell science
cDNA: Complementary DNA
MTT: [3′-(4,5-Dimethylthiazol-2-yl)-2,5-diphenyl tetrazolium bromide]
MCODE: Molecular complex detection
IHC: Immunohistochemistry
BM: Brain metastasis
PDAC: Pancreatic ductal adenocarcinoma
NK: Natural killer
