# Supplementary material for: Exploring the role of miR-200 family in regulating CX3CR1 and CXCR1 in lung adenocarcinoma tumor microenvironment: implications for therapeutic intervention
Source: Sci Rep. 2023 Sep 28;13:16333. doi: 10.1038/s41598-023-43484-1 (PMC10539366; doi:10.1038/s41598-023-43484-1)
Supplement: Supplementary file 1 — Supplementary Information. [file 41598_2023_43484_MOESM1_ESM.docx]

**Supplementary Information**

**Exploring the role of miR-200 family in regulating CX3CR1 and CXCR1 in lung adenocarcinoma tumor microenvironment: Implications for therapeutic intervention**

Archana Sharma**^1^**, Prithvi Singh**^2^**, Rishabh Jha**^2^**, Saleh A. Almatroodi**^3^**, Faris Alrumaihi**^3^**, Arshad Husain Rahmani**^3^**, Hajed Obaid Alharbi**^3^**, Ravins Dohare**^2,*^**, Mansoor Ali Syed**^1,*^**

**^1^**Translational Research Lab, Department of Biotechnology, Faculty of Natural Sciences, Jamia Millia Islamia, New Delhi 110025, India

**^2^**Centre for Interdisciplinary Research in Basic Sciences, Jamia Millia Islamia, New Delhi 110025, India

**^3^**Department of Medical Laboratories, College of Applied Medical Sciences, Qassim University, Buraydah 51452, Saudi Arabia

*** Correspondence**

| **Mansoor Ali Syed**, PhD  Assistant Professor  Department of Biotechnology, Faculty of Natural Sciences, Jamia Millia Islamia, New Delhi-110025  E-mail: [smansoor@jmi.ac.in](mailto:smansoor@jmi.ac.in)  Tel: +91-99537 86440 | **Ravins Dohare**, PhD  Assistant Professor  Centre for Interdisciplinary Research in Basic Sciences Jamia Millia Islamia, New Delhi-110025  E-mail: [ravinsdohare@gmail.com](file:///F:\miR-200\ravinsdohare@gmail.com)  Tel: +91-98686 55958 |
| --- | --- |


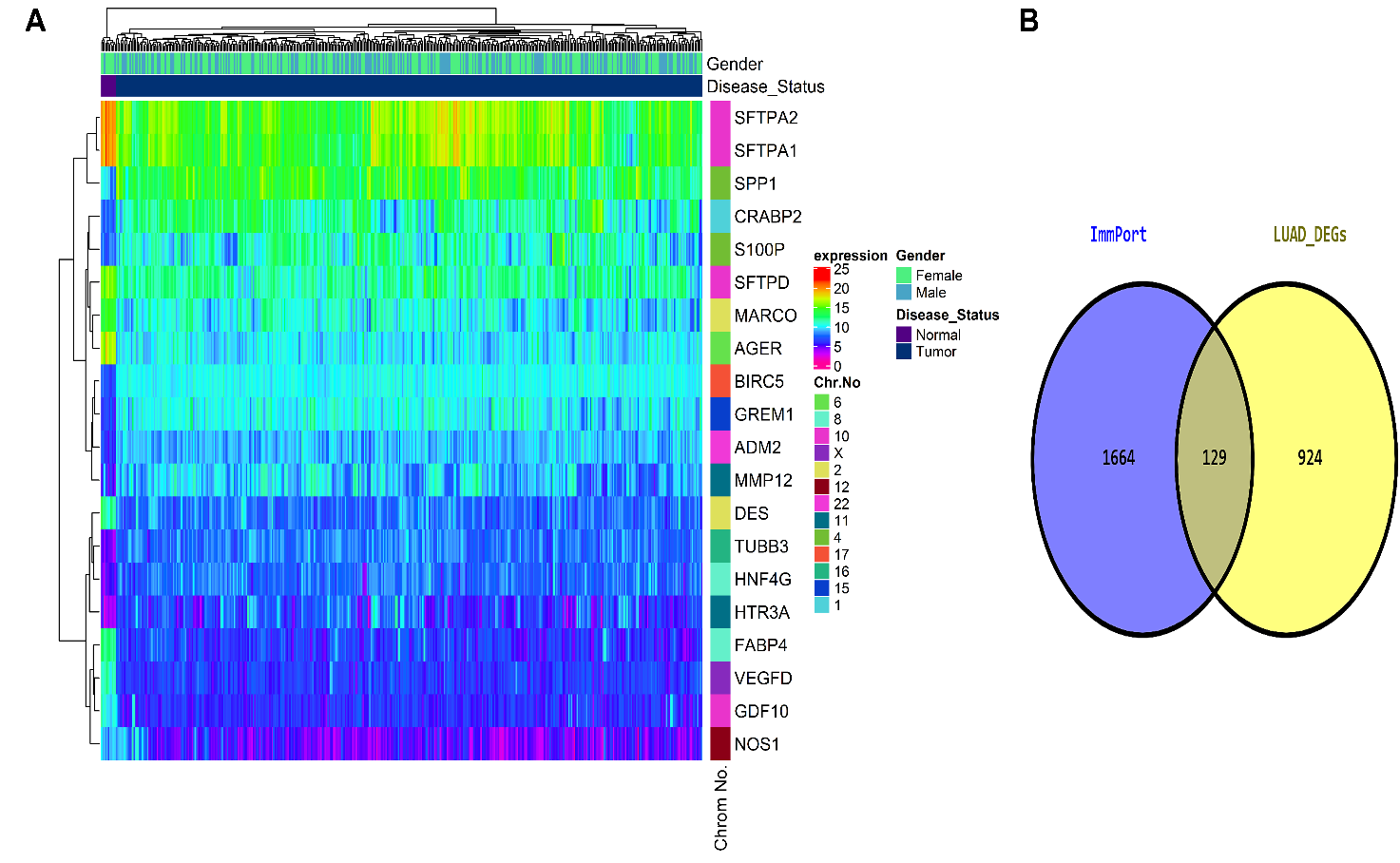
**Figure S1.** ***(A)*** *Annotation heatmap of top 10 up and downregulated LUAD-associated DEGs. Hierarchical clustering using Pearson distance was applied for both rows and columns with their corresponding cluster dendrograms displayed along the plot's left and top sides, respectively. The column annotation bars depicting disease status and gender of samples were shown at the top of the heatmap. The row annotation depicting location of gene on its corresponding chromosome was shown in right as a colored bar.* ***(B)*** *Venn plot showing the overlapping DEIRGs between LUAD-associated DEGs and ImmPort-obtained IRGs.*

*
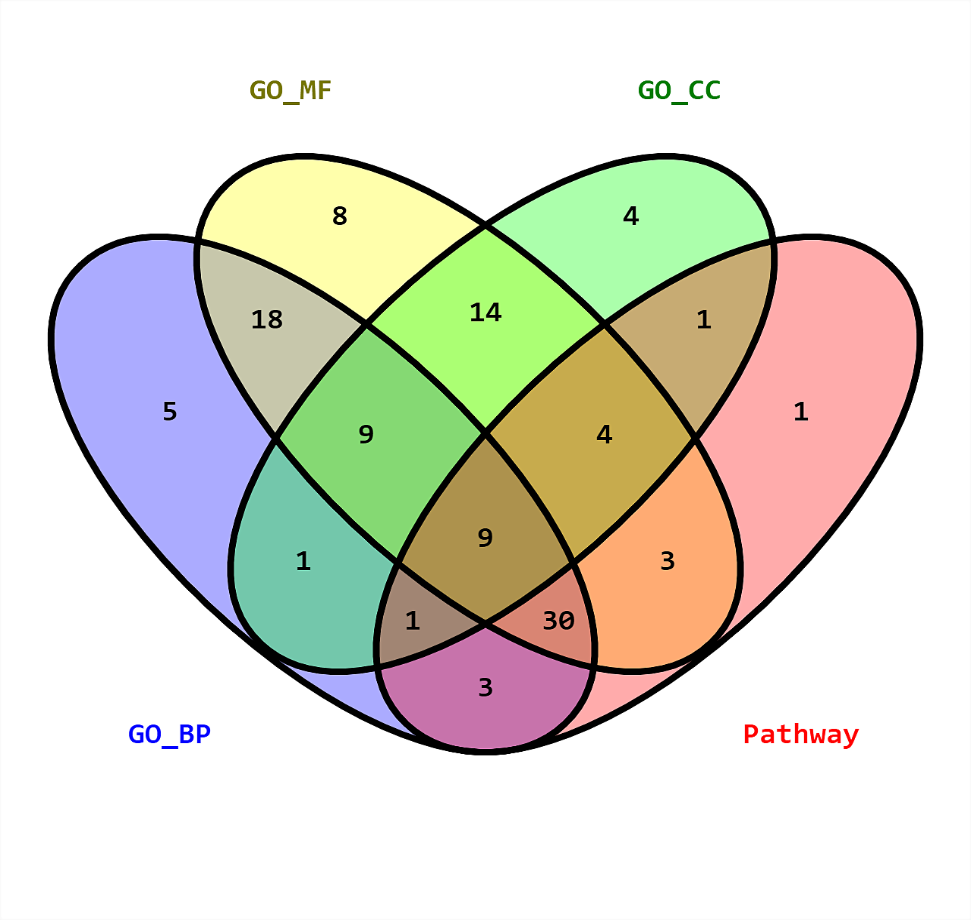
*

**Figure S2.** *Venn plot showing the overlapping genes between significantly enriched pathways and GO terms.*

**Table S1.** *Median OS time of FFL subnetwork motif items in lower and higher expression cohorts.*

| **Items** | **Low expression cohort (months)** | **High expression cohort (months)** |
| --- | --- | --- |
| miR-200a-3p | 34.87 | 54.07 |
| *CX3CR1* | 42.17 | 67.57 |
| *SPIB* | 41.17 | 57.5 |

***
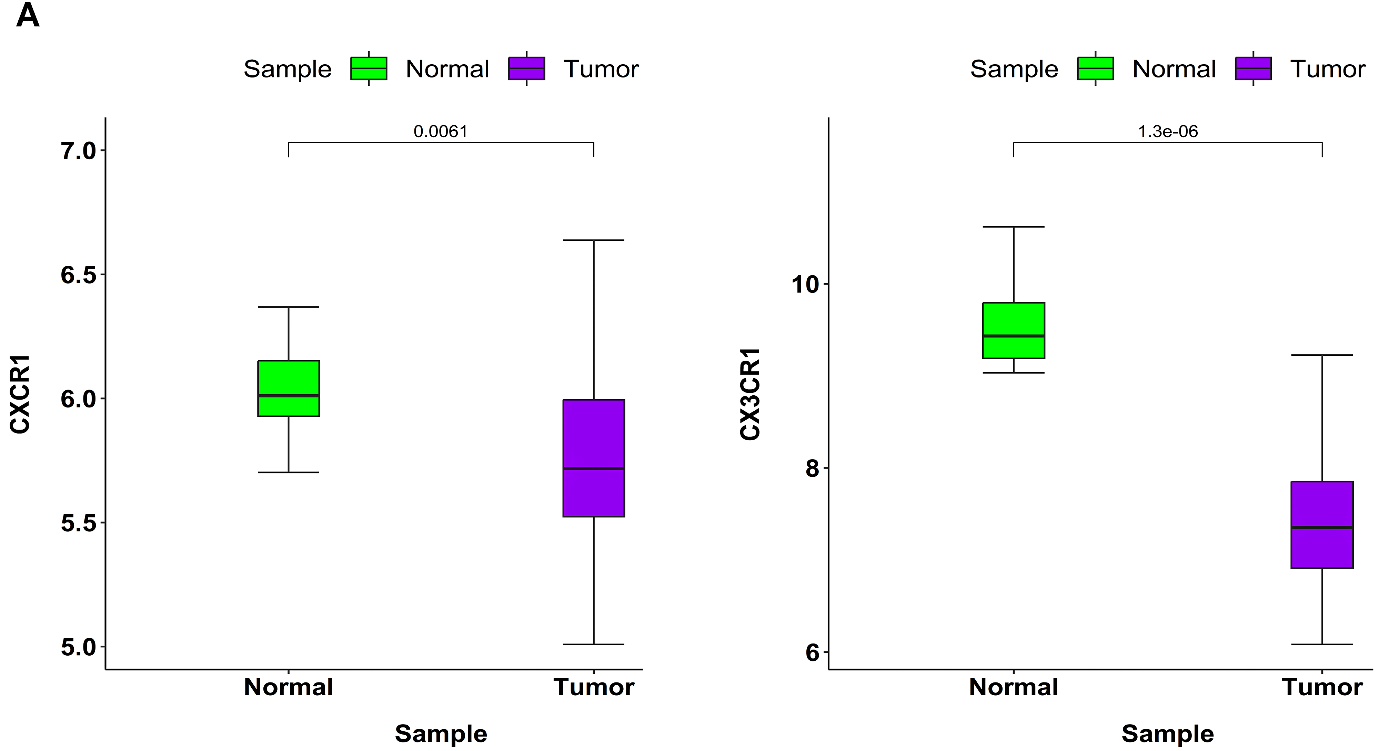
***

***
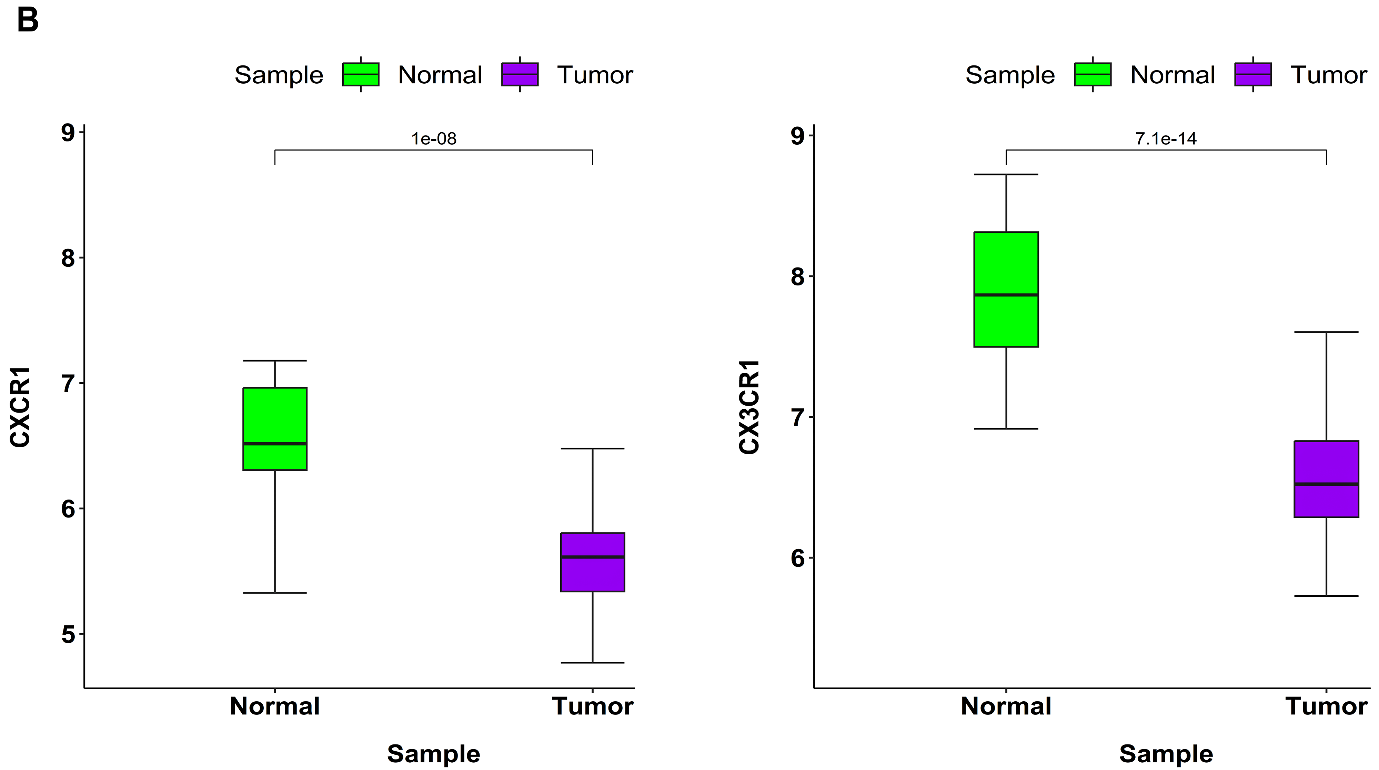
***

**Figure S3.** *Box-and-whisker plots showing mRNA expression distribution of CXCR1 and CX3CR1 across normal and LUAD samples in case of* ***(A)*** *GSE116959 and* ***(B)*** *GSE43458. Green- and magenta-colored boxes signify normal and tumor samples. The top and bottom of the boxes signify 75^th^ and 25^th^ percentile of distribution. Horizontal lines within the boxes represent the median values while minimum and maximum values label the axes endpoints.*
